# Are bio‐based resins suitable alternatives for additively manufactured removable dies? An in vitro study on dimensional and positional trueness and stability

**DOI:** 10.1111/jopr.70027

**Published:** 2025-09-08

**Authors:** Mustafa Borga Dönmez, Clara Lisa Soliva, Hanan Al‐Johani, Ahmet Orgev, Martin Schimmel, Gülce Çakmak, Burak Yilmaz

**Affiliations:** ^1^ Department of Prosthodontics Faculty of Dentistry Biruni University Istanbul Turkey; ^2^ Department of Reconstructive Dentistry and Gerodontology School of Dental Medicine University of Bern Bern Switzerland; ^3^ Department of Restorative Dentistry Faculty of Dentistry King Abdulaziz University Jeddah Saudi Arabia; ^4^ Department of Restorative Dentistry School of Dental Medicine University at Buffalo Buffalo New York USA; ^5^ Department of Prosthodontics Geriatric Dentistry and Craniomandibular Disorders Corporate Member of Freie Universität Berlin and Humboldt‐Universität zu Berlin Charité‐Universitätsmedizin Berlin Berlin Germany; ^6^ Department of Restorative Preventive and Pediatric Dentistry School of Dental Medicine University of Bern Bern Switzerland; ^7^ Division of Restorative and Prosthetic Dentistry The Ohio State University Columbus Ohio USA; ^8^ Department of Prosthodontics Faculty of Dentistry Ankara University Ankara Turkey

## Abstract

**Purpose:**

This study aimed to compare the dimensional and positional deviations of additively manufactured removable dies fabricated using two bio‐based resins and one conventional dental cast resin, while also evaluating these outcomes over a 4‐week period.

**Materials and Methods:**

A right mandibular first molar preparation on a typodont was scanned to digitally design removable dies and hollow partial arch casts. Based on a priori power analysis, a total of 30 dies (*n* = 10) and three hollow casts (*n* = 1) were fabricated using additive manufacturing (AM) from three different dental cast resins: DentaMODEL (DM), FotoDent bio‐based model (CB), and soy‐based resin (SB). The dies and their seated positions in casts were digitized 1 day (T0), 1 week (T1), 2 weeks (T2), 3 weeks (T3), and 4 weeks (T4) after fabrication. Dies’ dimensional deviations (crown, root, base of the root, and overall) and positional deviations in casts (crown region surface and point‐based) at T0 were defined as trueness, while deviations measured over 4 weeks (T0–T4) were defined as stability. The deviations measured at T0 were analyzed either using a generalized linear model (dimensional deviations) or one‐way analysis of variance (crown region and point‐based deviations). The deviations measured over the 4‐week period were analyzed with generalized linear model analysis and Bonferroni‐corrected post hoc tests (*α* = 0.05).

**Results:**

CB dies mostly had the lowest and SB dies mostly had the highest dimensional deviations (*p *≤ 0.001). The crown region had the lowest dimensional deviations, while the dimensional deviations measured at T3 were higher than those at T2 and T4 (*p *≤ 0.003). SB dies had the highest and CB dies had the lowest positional deviations, while crown region deviations were lower at T0 and T1 than at T4, and point‐based deviations were lower at T0 than at T4 (*p *≤ 0.049).

**Conclusions:**

CB dies mostly had better dimensional and positional trueness and stability over 4 weeks. The changes in tested outcomes for all dies over time were small.

Computer‐aided design and computer‐aided manufacturing (CAD‐CAM) technologies, along with intraoral scanners (IOSs), have enabled the digitalization of a patient's intraoral condition through a cost‐ and time‐efficient direct digital workflow.^1^, ^2^, ^3^ This digital workflow eliminates the need for conventional impressions, which can be inconvenient for patients, and stone casts, which are prone to deformation.^4^ However, for complex situations or when enhanced esthetics are required, CAD‐CAM technologies can also be used to fabricate physical casts with removable dies.^4^, ^5^ In this regard, additive manufacturing (AM) offers a promising alternative, allowing the efficient layer‐by‐layer fabrication of complex geometries, including removable dies, while reducing material waste.^6^, ^7^, ^8^, ^9^ Among AM technologies, vat polymerization, which is based on the photopolymerization of liquid resin, is commonly preferred for removable die and dental cast fabrication.^2^, ^9^, ^10^, ^11^

AM removable dies differ from those in dental stone as they are fabricated and postprocessed separately before being assembled with the definitive cast. Since the accuracy and fit of these dies are critical for fabricating precise restorations, understanding the factors influencing their dimensional stability is essential.^12^ One fundamental factor is the dental cast resin used, as it directly impacts fabrication accuracy and long‐term stability.^1^, ^13^, ^14^, ^15^, ^16^, ^17^ However, most resins available for 3D printers that operate on vat polymerization technology contain epoxide‐ or acrylate‐based resins derived from fossil resources, which contribute to an increased carbon footprint.^18^, ^19^ Recently, bio‐based resins exhibiting reduced toxicity and favorable characteristics have been developed,^19^, ^20^, ^21^, ^22^, ^23^, ^24^, ^25^ offering potential for use in the AM of dental appliances.^1^, ^11^, ^26^, ^27^ Integrating such resins into clinical and laboratory protocols may improve the environmental sustainability of AM, particularly if they demonstrate stable dimensions over time. Ensuring reusability is essential, as repeatedly producing identical casts and removable dies, regardless of the resin, poses ecological concerns.

Several recent studies have evaluated the fit and fabrication accuracy of AM removable dies.^4^, ^7^, ^8^, ^9^, ^15^, ^28^, ^29^, ^30^, ^31^, ^32^, ^33^, ^34^ However, only three have investigated their dimensional stability,^9^, ^32^, ^34^ and only one has specifically examined bio‐based dental cast resins.^34^ Since a portion of the chemical composition of bio‐based resins is derived from plants, this may influence the printability of the dies and casts, as well as their dimensional and positional stability. Dimensional inaccuracies in the dies can result in misfit within the casts, potentially compromising the accuracy of restorations fabricated or adjusted on these dies.^12^, ^15^ Given the promising findings from previous studies on the dimensional stability of AM casts using bio‐based dental cast resins,^1^, ^26^ further research into the various applications of these materials is warranted. Such investigations could enhance the understanding of their limitations among clinicians and dental technicians while promoting their adoption in more environmentally sustainable clinical practices. Therefore, the present study aimed to compare both the dimensional and positional deviations of AM removable dies fabricated from two bio‐based dental cast resins against a conventional dental cast resin. These outcomes were evaluated immediately after fabrication and over a 4‐week period. The null hypotheses were that (i) the type of dental cast resin and the region on the removable die would not affect the dimensional trueness; (ii) the type of dental cast resin would not affect the positional trueness; (iii) the type of dental cast resin, the region on the removable die, and the time point would not affect the dimensional stability; and (iv) the type of dental cast resin and the time point would not affect the positional stability of the tested dies.

## MATERIALS AND METHODS

**FIGURE 1:**
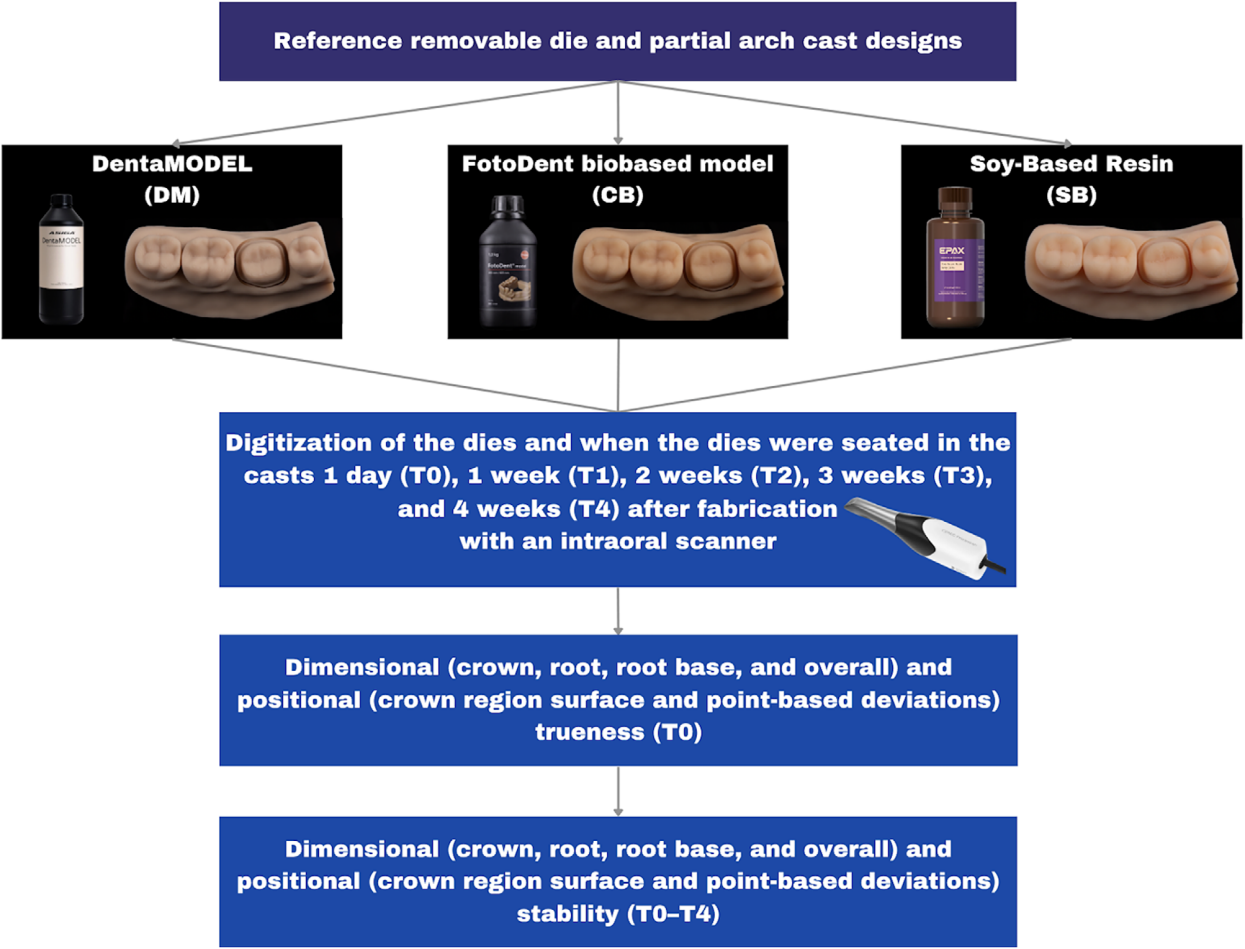
Overview of study design.

Figure 1 presents the overview of this study. A typodont mandibular right first molar in a dentate model (ANA‐4; Frasaco GmbH) was prepared with a chamfer finish line measuring 1 mm in width. A partial arch scan was made using an IOS (CEREC Primescan v5.2; Dentsply Sirona). The scan data were exported as a standard tessellation language (STL) file and imported into a CAD software program (DentalCAD 3.0 Galway; exocad GmbH), where the model creator function was used to design a hollow cast with a removable die section. The removable die was designed with the following parameters: 1.5 mm pin height, 0 mm preparation margin extrusion, 1 mm seating width, 10 mm taper height, and 0° shaft taper angle. The hollow cast parameters varied depending on the resin used: a 0.08‐mm horizontal and vertical shaft gap for conventional (DentaMODEL [DM]; Asiga) and corn‐based (FotoDent biobased model [CB]; Dreve Dentamid GmbH) dental cast resins, and a 0.2‐mm gap for the soy‐based dental cast resin (soy‐based resin [SB]; EPAX). These parameters were determined through pilot testing to ensure proper seating of the dies without resistance. Additional parameters included a 0‐mm pedestal height, 0.5‐mm average ditch width and depth, 2‐mm average ditch height, 2.5‐mm wall thickness, and a 1‐mm cavity fill diameter.^4^, ^8^ After finalizing the designs, the reference STL files, including those for the removable dies (D‐STL), hollow partial arch casts with the die (PWD‐STL), and without the die (PWOD‐STL), were exported for subsequent processing.

For fabrication, the D‐STL and PWOD‐STL files were individually imported into separate build jobs using nesting software (Composer v2.0; Asiga). Within each job, both STL files were oriented with their bases parallel to the build platform. After automatic generation of support structures, the D‐STL file was duplicated to position a total of 10 dies on the platform. The nesting data for each set of STLs were then imported into a digital light processing (DLP)‐based 3D printer (MAX UV; Asiga). Using a consistent layer thickness of 100 µm, a total of 30 removable dies (*n* = 10) and three hollow casts (*n* = 1) were produced from three different dental cast resins (Table 1). The sample size was calculated through power analysis, using parameters of *α* = 0.05, 1 − *β* = 95%, and an effect size of *f* = 0.623, based on the findings from a previous study.^8^

**TABLE 1:** List of tested dental cast resins.

Material	Abbreviation	Chemical composition	Viscosity (mPa s)
DentaMODEL	DM	7,7,9(or 7,9,9)‐trimethyl‐4,13‐dioxo3,14‐dioxa‐5,12‐ diazahexadecane‐1,16‐diyl bismethacrylate: 10%–25%	400
		Tetrahydrofurfuryl methacrylate: 10%–20%	
		Diphenyl(2,4,6‐trimethylbenzoyl) phosphine oxide: <1%	
FotoDent bio‐based model	CB	Acrylic oligomer: ≥50% Fatty acids, C18‐unsatd., dimers, polymers with acrylic acid and 1,3,5‐tris(2‐hydroxyethyl)−1,3,5‐triazine‐2,4,6(1H,3H,5H)‐trione: ≥1–<10%	600
Soy‐based resin	SB	Acrylic oligomers: 40%–50%	400–500
		Monomer: 20%–40%	
		Color pigments: 2%–5%	
		Photoinhibitors: 3%–5%	

**FIGURE 2:**
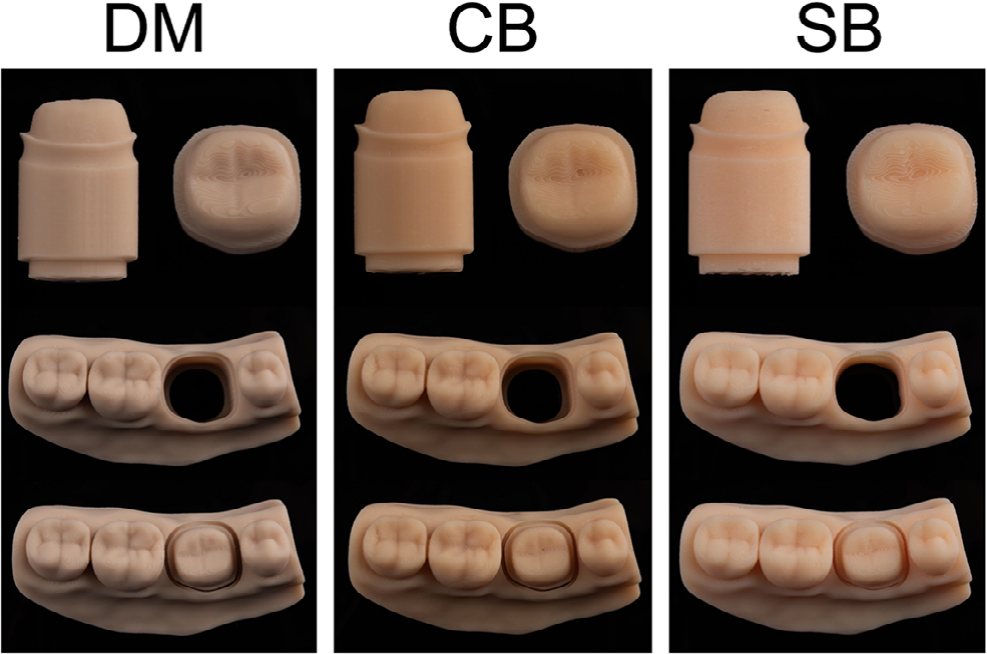
Representative images of removable dies and partial arch casts for each tested resin. CB, Fotodent bio‐based model resin; DM, DentaModel; SB, soy‐based resin.

After fabrication, all removable dies and partial arch casts were ultrasonically cleaned in isopropyl alcohol (DM and CB casts: 2 × 5 min; SB casts: 2 × 6 min) and postpolymerized in a nitrogen oxide gas atmosphere using a xenon polymerization unit (Otoflash G171; NK Optik GmbH) for 4000 light exposures (2 × 2000 flashes) (Figure 2). The test STL files of the removable dies (TD‐STLs) and the dies seated within their respective hollow casts (PWDT‐STLs) were generated using the same IOS 1 day (T0) after the fabrication to minimize dimensional changes.^4^, ^8^ The process was repeated at 1‐week (T1), 2‐week (T2), 3‐week (T3), and 4‐week (T4) intervals postfabrication following the same protocol. All scans were performed by one operator (C.S.), with the IOS calibrated prior to each session. Scanning took place under standardized environmental conditions, including controlled temperature (23°C), and ambient humidity and lighting. To minimize the risk of dimension changes due to environmental exposure, all dies and casts were stored in light‐proof boxes within the same room between intervals.^33^

**FIGURE 3:**
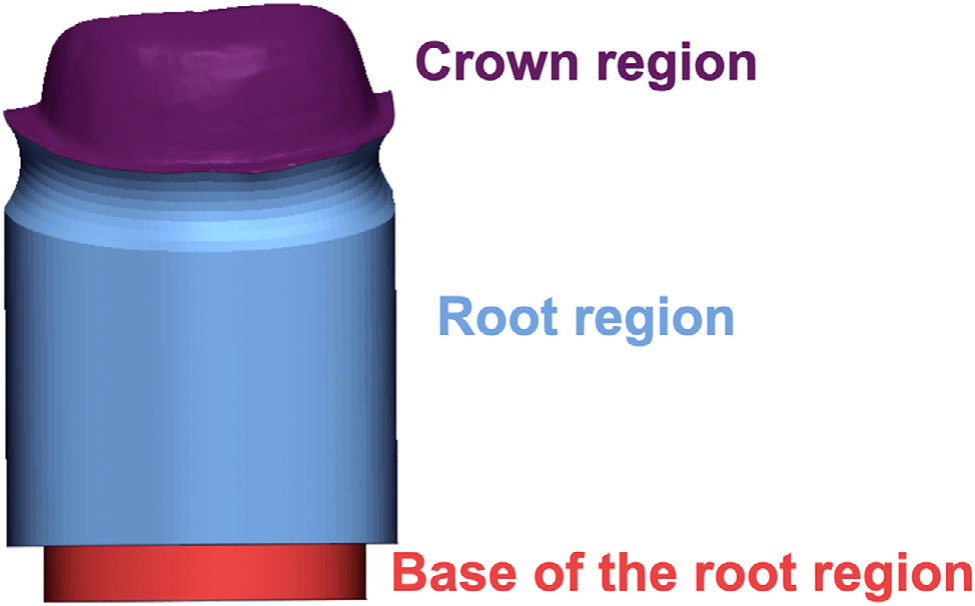
Virtually segmented D‐STL file. D‐STL: reference removable die standard tessellation language file.

**FIGURE 4:**
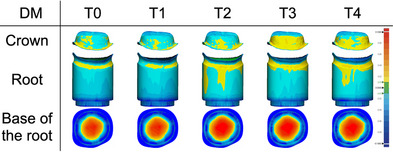
Representative color maps of different regions of DM removable die. DM, DentaMODEL resin; T0, 1 day after fabrication; T1, 1 week after fabrication; T2, 2 weeks after fabrication; T3, 3 weeks after fabrication; T4, 4 weeks after fabrication.

**FIGURE 5:**
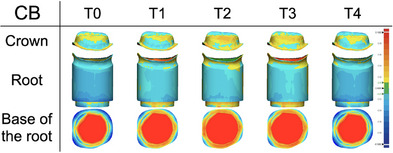
Representative color maps of different regions of CB removable die. CB, FotoDent bio‐based model resin; T0, 1 day after fabrication; T1, 1 week after fabrication; T2, 2 weeks after fabrication; T3, 3 weeks after fabrication; T4, 4 weeks after fabrication.

**FIGURE 6:**
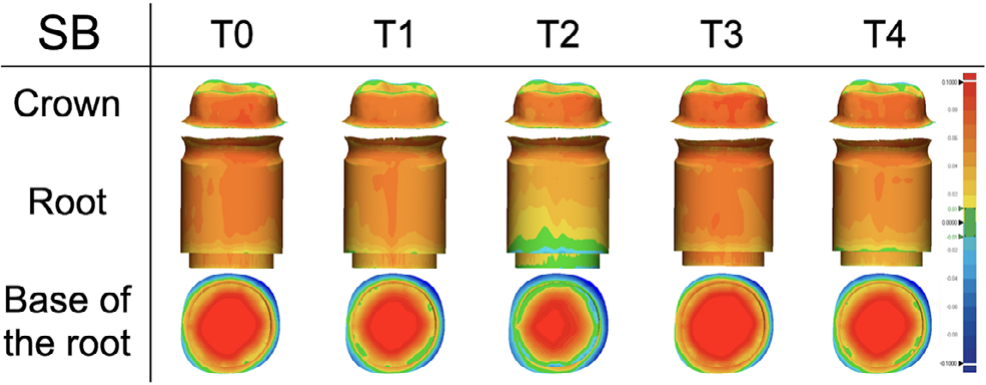
Representative color maps of different regions of SB removable die. SB, soy‐based model resin; T0, 1 day after fabrication; T1, 1 week after fabrication; T2, 2 weeks after fabrication; T3, 3 weeks after fabrication; T4, 4 weeks after fabrication.

After completing the scans, all STL files were imported into metrology‐grade 3D analysis software (Geomagic Control X v2022.1.1; 3D Systems) for dimensional stability analyses. The D‐STL files were first imported into the software, where the region tool was used to divide each die into three anatomical segments: crown, root, and root base (Figure 3), enabling both localized and overall deviation assessments. Following segmentation, each time‐specific TD‐STL was superimposed onto its corresponding D‐STL using a combination of initial and iterative closest point best‐fit alignment algorithms. The software automatically computed root mean square (RMS) values to measure deviations from the D‐STLs at each time point. For qualitative analysis, color maps were generated with a tolerance of ±10 µm and nominal values of ±100 µm using the 3D Compare tool (Figures 4, 5, 6). Additionally, the same tool was used to isolate the crown region of the removable die and adjacent teeth in each PWD‐STL. Each corresponding PWDT‐STL was then aligned over its segmented PWD‐STL using the same alignment algorithms, excluding the crown region. RMS values for the adjacent teeth were calculated for each PWDT‐STL and averaged to evaluate the influence of the 3D printer and IOS on deviations in the partial arch casts with dies.

**FIGURE 7:**
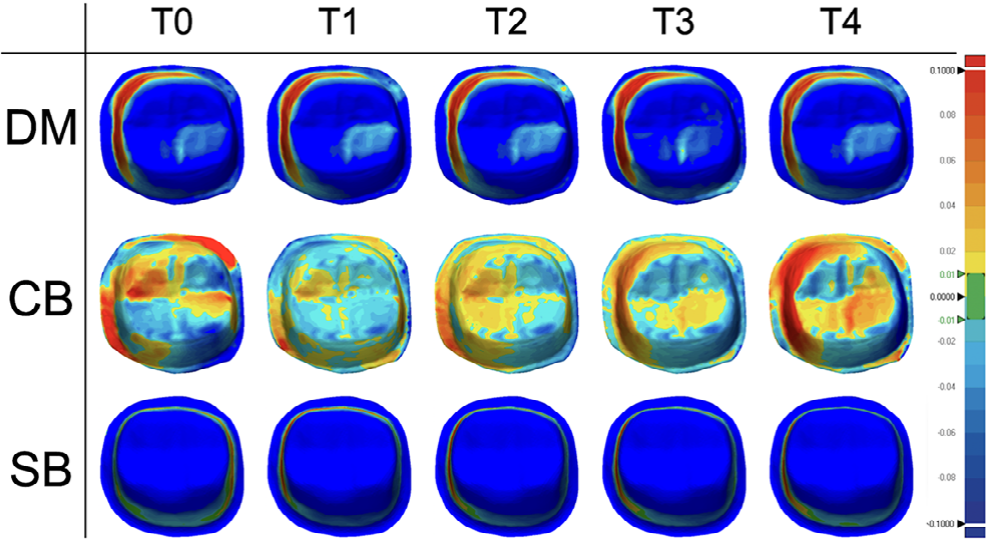
Representative color maps of crown region of dies seated in partial arch casts. CB, Fotodent bio‐based model resin; DM, DentaModel; SB, soy‐based resin; T0, 1 day after fabrication; T1, 1 week after fabrication; T2, 2 weeks after fabrication; T3, 3 weeks after fabrication; and T4, 4 weeks after fabrication.

**FIGURE 8:**
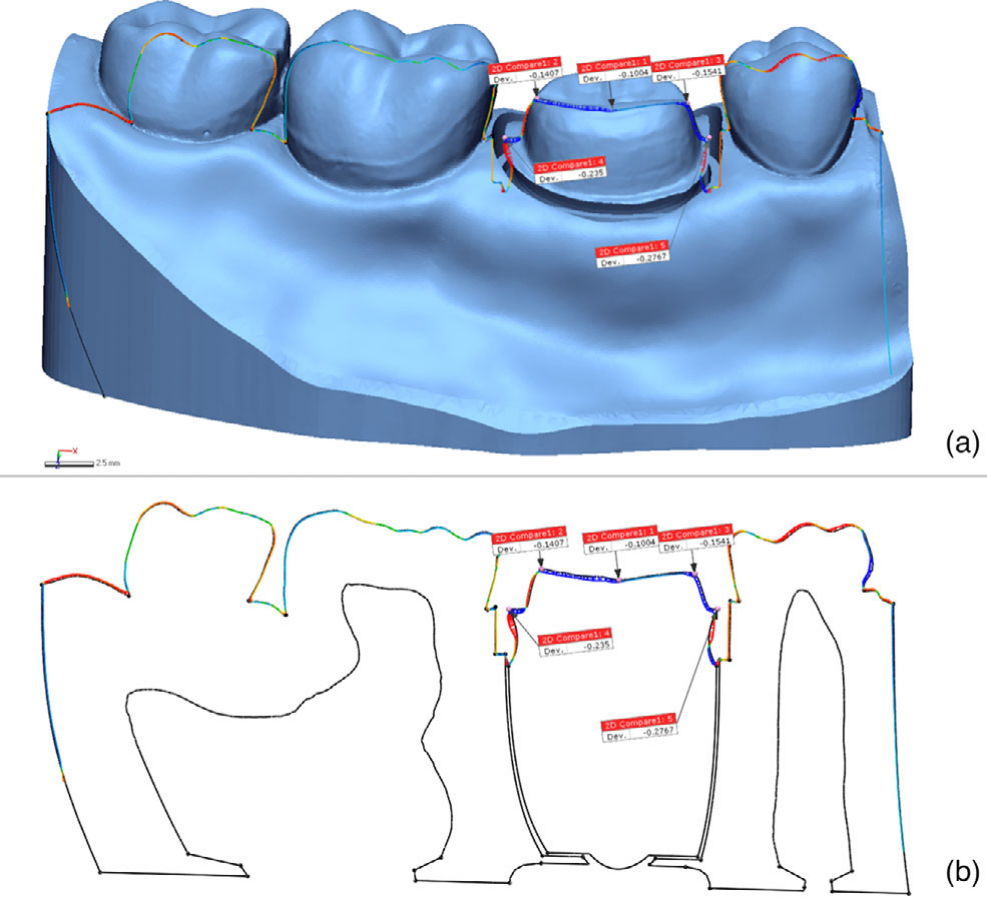
Point‐based fit measurement of removable dies. (a) Points generated for fit measurement; (b) cross‐sectional view.

Positional deviations of the removable dies were quantitatively assessed by analyzing surface deviations in the crown region of the partial arch cast, alongside a previously applied point‐based measurement method. Qualitative analysis was performed using color maps to visualize the crown region of the seated dies (Figure 7). For the point‐based analysis, a two‐dimensional plane was created along the mesiodistal axis of the crown region of the die. Five key points were selected on the PWD‐STL and PWDT‐STL files: the most distal point of the margin, the tip of the distal cusp, the deepest point of the occlusal fossa, the tip of the mesial cusp, and the most mesial point of the margin (Figure 8). The software automatically calculated the distance deviations between these points, recording them as negative or positive values. A negative value indicated that the point on the PWDT‐STL was below its counterpart on the PWD‐STL, while a positive value indicated the opposite. Absolute values of these deviations were used for statistical analysis.^4^, ^8^ All analyses were performed by one experienced prosthodontist (G.Ç.).

The deviations measured at T0 were used to determine the dimensional and positional trueness of the tested dies, while deviations measured at consecutive time points defined their dimensional and positional stability. Data distribution was analyzed with the Shapiro Wilk test, and the homogeneity was analyzed with the Levene test. At T0, the dimensional deviations of the dies, crown region deviations in the partial arch cast, and point‐based deviations showed a normal distribution. Therefore, dimensional deviations were analyzed using a generalized linear model with an identity function, while crown region and point‐based deviations were analyzed using one‐way analysis of variance. The dimensional and positional deviations over the 4‐week period demonstrated a non‐normal distribution and were analyzed using generalized linear model analyses with a gamma distribution and log link. Further analyses were performed with Bonferroni‐corrected post hoc tests. All statistical analyses were conducted using a software program (SPSS v29.0; IBM Corp) at a significance level of *α* = 0.05.

## RESULTS

Dimensional deviations at T0 were significantly influenced by resin type, die region, and their interaction (*p *< 0.001). Additionally, resin type had a significant effect on both crown region deviations in the cast and point‐based deviations (*p *< 0.001). The interaction between resin type and time point, resin type and die region, as well as each main factor, significantly influenced the dimensional deviations of removable dies over time (*p *< 0.001). Positional deviations (both crown region surface and point‐based deviations) over time were affected by resin type (*p *< 0.001) and time point (*p *≤ 0.044), but the interaction between these factors did not significantly impact positional trueness (*p *≥ 0.074).

When considering clinically relevant comparisons for the dimensional deviations measured at T0, SB dies had the highest crown region deviations, CB dies had the lowest root region and overall deviations, and DM dies had the highest base of the root region deviations (*p *< 0.001). The measured deviations of different die regions were listed in ascending order as crown region, overall, root region, and base of the root region for both DM and CB dies (*p *≤ 0.003). However, within SB dies, the crown region had the lowest deviations and overall deviations were lower than those of the base of the root region (*p *≤ 0.003, Table 2). CB dies had the lowest crown region deviations and point‐based deviations, while the crown region deviations of DM dies were lower than SB dies (*p *< 0.001, Table 3).

**TABLE 2:** Mean dimensional deviation values and standard deviations (µm) measured at T0.

	Resin type		
Region	DM	CB	SB
Crown	20.6 + 1.5^a^	17.8 + 1.4^a^	45.7 + 3.9^c^
Root	61.7 + 5.0^f^	42.1 + 2.3^c^	56.7 + 4.2^ef^
Base of the root	104.6 + 8.1^g^	67.5 + 4.8^f^	64.7 + 5.3^f^
Overall	47.4 + 2.9^cd^	34.4 + 1.9^b^	53.3 + 3.9^de^

*Note*: Different superscript lowercase letters indicate significant differences among resin‐region pairs (*p *< 0.05).

**TABLE 3:** Mean and standard deviation crown region surface deviations (µm) and median point‐based deviations (µm) of seated dies measured at T0.

Resin type	Crown region deviations	Point‐based deviations
DM	129.9 + 9.3^b^	212.4 + 27.2^b^
CB	38.9 ± 10.1^a^	44.3 + 9.4^a^
SB	207.6 + 3.0^c^	280.8 + 8.2^c^

*Note*: Different superscript lowercase letters indicate significant differences among resins (*p* < 0.05).

When the dimensional deviations of dies over time were considered, CB dies had the lowest deviations in all regions except for the base of the root, where SB dies had the lowest deviations (*p *< 0.001). SB dies had the highest crown region and overall deviations (*p *< 0.001). Regardless of the resin type, the crown region had the lowest and the base of the root region had the highest deviations, followed by the root region (*p *≤ 0.016). Within each time point, CB dies had the lowest and SB dies had the highest dimensional deviations (*p *< 0.001). DM dies had higher deviations at T1 than at T2 and T3 (*p *≤ 0.019). CB dies had higher deviations at T2 than at T4 (*p* = 0.030), while SB dies had the highest deviations at T3 (*p *≤ 0.001, Table 4).

**TABLE 4:** Gamma‐adjusted means of region‐based and overall dimensional deviations over 4‐week period (µm) (raw mean ± standard deviation).

	Resin type		
Region	DM	CB	SB
Crown	20.9^b^	17.2^a^	46.4^e^
	(20.9 + 1.6)	(17.2 + 1.6)	(46.4 + 4.0)
Root	59.9^g^	40.7^d^	57.5^g^
	(60.0 + 5.6)	(40.7 + 2.3)	(57.5 + 5.7)
Base of the root	100.7^J^	74.8^i^	67.3^h^
	(100.8 + 9.9)	(75.0 + 8.3)	(67.4 + 8.4)
Overall	46.3^e^	32.7^c^	54.0^f^
	(46.4 + 3.6)	(32.8 + 1.7)	(54.1 + 5.1)
**Time point**			
T0	50.0^CDE^	36.3^AB^	54.6^FG^
	(58.5 + 31.1)	(40.4 + 18.3)	(55.1 + 8.1)
T1	51.7^DEF^	35.4^AB^	55.7^G^
	(60.5 + 32.2)	(40.8 + 22.0)	(56.2 + 9.2)
T2	47.7^C^	37.5^B^	52.6^EFG^
	(54.7 + 27.9)	(43.6 + 24.4)	(53.0 + 8.8)
T3	48.2^C^	36.8^AB^	60.7^H^
	(55.4 + 28.5)	(42.6 + 23.5)	(61.3 + 10.0)
T4	48.4^CD^	35.0^A^	55.8^G^
	(55.9 + 29.0)	(39.7 ± 2 0.1)	(56.3 + 10.4)

*Note*: Different superscript lowercase letters indicate significant differences among resin‐region pairs, while different superscript uppercase letters indicate significant differences among resin‐time point pairs (*p* < 0.05).

**FIGURE 9:**
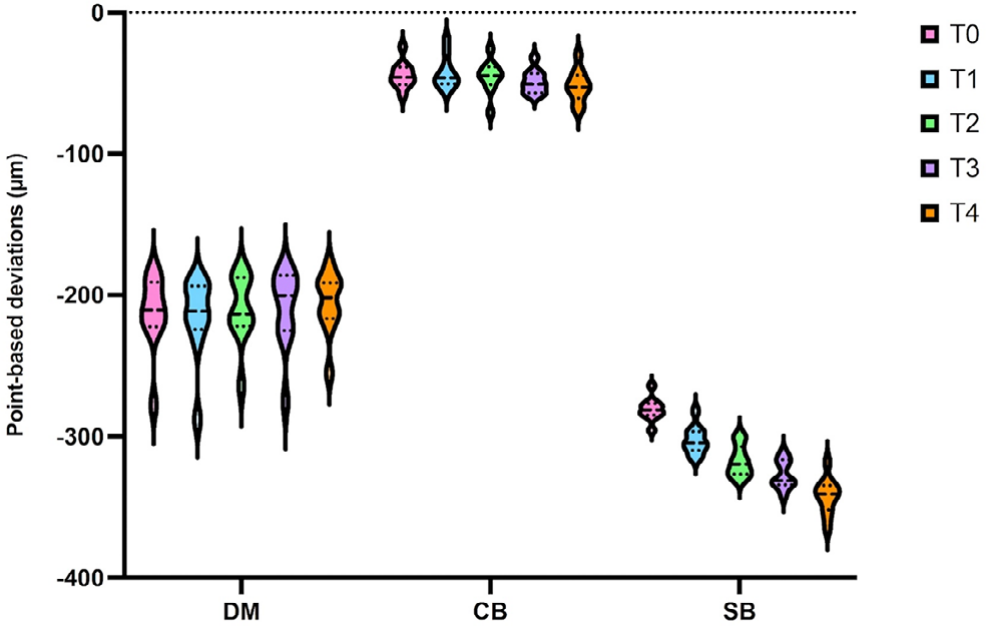
Distribution of raw point‐based distance measurement data. CB, Fotodent bio‐based model resin; DM, DentaModel; SB, soy‐based resin; T0, 1 day after fabrication; T1, 1 week after fabrication; T2, 2 weeks after fabrication; T3, 3 weeks after fabrication; and T4, 4 weeks after fabrication.

When examining positional deviations of the dies over time, SB dies exhibited the highest crown region and point‐based deviations, regardless of the time point (*p *< 0.001). CB dies demonstrated the lowest crown region and point‐based deviations (*p *< 0.001). The removable dies exhibited lower crown region deviations at T0 and T1 compared to T4 (*p *≤ 0.012). Bonferroni‐corrected post hoc tests indicated that the differences in point‐based deviations were lower at T0 than at T4 (*p* = 0.049, Table 5). Table 6 presents the RMS values for partial‐arch dentate casts for each resin‐time point combination, while Figure 9 displays the distribution of the raw point‐based deviation values.

**TABLE 5:** Gamma adjusted means of crown region surface deviations (µm) and point‐based deviations (µm) of seated dies over 4‐week period (raw mean ± standard deviation).

Resin type	Crown region deviations	Point‐based deviations
DM	129.7^B^	210.7^B^
	(129.7 ± 8.8)	(210.7 ± 25.6)
CB	41.4^A^	46.6^A^
	(41.6 ± 9.8)	(46.7 ± 10.7)
SB	224.4^C^	313.3^C^
	(224.7 ± 12.1)	(314.0 ± 23.5)
**Time point**		
T0	101.5^a^	138.3^a^
	(125.4 ± 70.5)	(179.2 ± 102.4)
T1	101.8^a^	140.6^ab^
	(128.9 ± 75.9)	(186.9 ± 111.4)
T2	107.0^ab^	144.7^ab^
	(132.9 ± 7.7)	(191.0 ± 115.0)
T3	107.9^ab^	149.9^ab^
	(133.7 ± 78.2)	(195.3 ± 117.2)
T4	114.4^b^	154.0^b^
	(139.0 ± 80.2)	(200.0 ± 121.6)

*Note*: For each outcome, different superscript uppercase letters indicate significant differences among resin types and different superscript lowercase letters indicate significant differences among time points (*p* < 0.05).

**TABLE 6:** Overall mean dimensional deviation values (µm) of dentate partial arch casts without die over 4‐week period.

	Resin type		
Time point	DM	CB	SB
T0	46.1	57.1	71.4
T1	46.8	55.7	70.2
T2	44.3	55.6	73.8
T3	44.1	56.6	74.6
T4	44.6	56.9	73.6

## DISCUSSION

DM and CB dies had the highest dimensional trueness, whereas the base of the root region of DM dies had the lowest trueness. SB dies had the lowest and CB dies had the highest positional truenesswhen the dies were seated in casts. Regardless of the time point, CB dies mostly had the lowest deviations within the tested die regions, indicating their high dimensional stability, whereas the opposite trend was observed for SB dies. In addition, the crown region of the tested dies exhibited the highest dimensional stability, while the base of the root region showed the lowest dimensional stability. DM dies demonstrated greater dimensional stability at T2 and T3 compared to T1. SB dies had higher stability at T2 than at T4, and CB dies exhibited the lowest stability at T3. Within each time point, SB dies had the highest and CB dies had the lowest stability. CB dies had the highest and SB dies had the lowest positional stability, regardless of the time point. Additionally, tested dies had higher positional stability, as indicated by crown region and point‐based deviations, at T0 compared to T4. Therefore, all null hypotheses were rejected.

The accuracy of fixed restorations depends on the dimensional trueness of removable dies and their positional trueness within the corresponding dentate casts. The dimensional trueness and stability of AM cast resins are influenced by their composition and viscosity,^16^, ^35^ as well as AM technologies, printing parameters, and postprocessing conditions.^2^, ^6^, ^16^, ^35^, ^36^ In this study, AM removable dies and their corresponding partially dentate casts were fabricated in accordance with the respective manufacturers’ recommendations, digitized using the same IOS, fabricated with standardized printing parameters on a single DLP‐based 3D printer, and stored under identical conditions. However, slight disparities were present in the design of the hollow casts, as larger horizontal and vertical shaft gaps (0.2 mm) were implemented for the SB casts compared to the smaller gaps (0.08 mm) used for the DM and CB casts. These parameters were determined through pilot testing to enable resistance‐free seating, reflecting the distinct physical properties and curing behavior of the SB resin. Consequently, variations in measured dimensional deviations among the tested dies may be attributed primarily to the compositional differences in tested resins, while differences in positional deviations may also have been influenced by variations in shaft gap, resin formulation, and time point. The observed higher positional deviations, despite the use of a more permissive design, may highlight potential concerns regarding the positional stability of the SB resin for the tested indication. It is also important to acknowledge that using different design parameters introduces a variable that restricts direct comparison of intrinsic material behaviors. However, the objective was to evaluate each resin under conditions that reflect its practical, clinical use, rather than imposing a uniform design that might reduce clinical relevance for some materials. In addition, all dies were digitally assessed against their own respective design files, which served as the controls, thereby minimizing the influence of design differences on the trueness and fit evaluations.

The qualitative assessment of the demonstrative color maps could support the quantitative dimensional deviation findings of the removable die‐dentate cast complex across each time point. In color maps, red indicates overcontoured surfaces, blue represents undercontoured surfaces, and green signifies minimal deviations that are acceptable regardless of direction. Although statistically significant differences were observed among the tested AM resins within the same die regions, the actual magnitude of these differences remained relatively small. The greatest mean dimensional deviation was 33.4 µm, observed at the base of the root region between DM and SB resins. Similarly, the largest change over time within the same resin was 8.3 µm for SB between T2 and T3. Although these differences reached statistical significance, they fall well below commonly cited clinical thresholds for acceptable dimensional deviation in AM casts, which range from 120^10^ to 250 µm.^13^ Given the absence of specific clinical benchmarks for removable dies, these cast‐based thresholds provide a reasonable reference, supporting the interpretation that the observed deviations are likely clinically negligible despite being statistically significant. Nevertheless, CB dies exhibited the highest dimensional stability across the 4‐week observation period in all regions, except for the base of the root region. This was reflected in their color maps, which showed yellow and light blue zones along the crown region and predominantly light blue in the root region, indicating slight degrees of over‐ and undercontouring. The superior dimensional stability of this bio‐based resin was also reported in previous studies on AM casts, where it was attributed to its longer polymerization time compared to other cast resins.^1^, ^27^ However, in the present study, polymerization time was standardized across all tested groups. Therefore, the favorable dimensional stability of CB dies may be attributed to the resin's chemical composition, particularly the high ratio of acrylic oligomers (≥50%), which likely stabilized the polymerization reaction by strengthening the cross‐link network.^37^ Red was detected along the base of the root region, indicating an outward deviation that may have resulted from the manual support removal. This was also observed in the color maps of the base of the root regions across the other resin groups to varying degrees. In contrast, the SB dies showed findings opposite to those of the CB dies, with significantly higher crown region and overall dimensional deviations, as confirmed by the predominant orange and red in their color maps. The overcontoured SB surfaces remained consistent across all time points, suggesting the potential need for extensive adjustments to prevent geometrical distortion of the restorations adjusted on them. Considering the similar viscosity of tested resins, this could be attributed to the distinct chemical composition of the SB resin. Although the manufacturer has partially disclosed the chemical composition of the SB resin, its fatty acid content remains unknown. Considering that fatty acids can reduce cross‐link density and increase structural flexibility, which may contribute to greater deformation,^38^ the significantly higher deviations observed in the SB resin may suggest that it contains more fatty acids than the CB resin. However, without detailed information on the resin formulations and the degree of conversion for both bio‐based resins, a precise explanation for the differences in dimensional stability cannot be provided. Further studies evaluating the chemical composition and degree of conversion of CB and SB resins are needed to better relate these findings to their material formulations. In terms of the DM dies, higher dimensional deviations were observed at T1 compared to the succeeding time points. The initial undercontouring of DM could be related to the polymerization shrinkage, which stabilized over time.^14^ This was confirmed by the DM color maps, which displayed blue zones of different shades along the root region and emerging yellow zones along the crown region, gradually increasing after the 2‐week storage period.

The positional deviations of the AM removable dies were quantitatively assessed through surface deviation values and point‐based analyses of the crown region of the seated dies, complemented by qualitative analysis of the color maps of the crown region within their corresponding partially dentate casts. Seated SB dies had the highest and seated CB dies had the lowest crown region surface deviations. Additionally, both DM and SB dies showed greater deviations compared to their corresponding dentate casts, with seated DM and SB dies’ crown region deviations exceeding the clinically acceptable threshold for prosthetic applications (>120 µm).^10^ Although the crown region deviations may have been influenced by the dimensional deviations of partial arch casts, and the impact of the dies' positioning within their casts on the occlusal and interproximal contacts of the definitive prostheses may be less significant, these inharmonious dimensional changes in DM and SB die and cast complexes could still impair the adjustment of occlusal and interproximal contacts on the restorations seated on these dies. Nevertheless, it should also be emphasized that the difference between the mean crown region deviations of the DM dies and the clinically acceptable threshold for prosthetic applications was only 9.9 µm, which is relatively minor and potentially clinically negligible. The crown region of seated DM dies displayed predominantly dark blue on the occlusal surfaces, with red areas on the proximal crown surfaces, indicating a decreased height and horizontal overcontouring, which might impair the fit of the restoration to be adjusted. In contrast, the crown region of the seated SB dies predominantly showed dark blue throughout the entire storage period, indicating undercontouring that could compromise the stability of the restoration once seated, potentially resulting in incorrect adjustments due to this instability. The color maps of the crown region of seated CB dies displayed a mix of blue and yellow zones from T0 to T3, with red zones becoming prominent at T4, suggesting the need for additional adjustments for dies stored longer than a month. Restorations placed on CB dies might also experience seating issues due to the overcontoured areas, indicated by yellow, orange, and red on the color maps. However, once the correct fit is established, restorations may require fewer adjustments due to the synchronized dimensional changes in the CB die and cast complexes. Similarly, the point‐based positional analysis corroborated the surface deviation findings in the crown region, showing that the CB dies exhibited the best fit, followed by the DM dies. Although the point‐based method is often regarded as more reliable, making it preferable for accurately quantifying surface deviations,^39^ the selection of measurement points was restricted to a two‐dimensional plane along the mesiodistal cross‐section of the die. This limitation could explain the discrepancies observed between the surface deviation and point‐based deviation measurements. Nevertheless, the mean point‐based deviations of the DM and CB dies in terms of positional stability remained below the clinically acceptable thresholds of 120^10^ or 250 µm,^13^ and may therefore be considered clinically acceptable. However, interpreting the raw point‐based data can also offer additional insight into the spatial positioning of the dies within their respective dentate casts, where negative values indicate apical displacement and positive values reflect coronal displacement. A clear tendency for apical positioning, with varying magnitudes, was observed across all dies, regardless of the time point. Disparities in vertical alignment among tested dies may be associated with the variations related to manual support removal, influenced by the varying viscosities of tested resins. Based on the fit results, particularly those of SB dies, it is crucial to assess the seating of the tested removable dies to ensure their applicability, as adjustments or even remakes may be necessary. The correct positioning of AM removable dies can be easily verified using verification matrices to detect vertical displacement and confirm interocclusal relationships.^12^ This would enable the required adjustments, preventing premature contacts of the seated restoration during maximum intercuspation and eccentric movements.

To the authors’ knowledge, only one study has examined the dimensional stability of AM removable dies made from the CB resin, comparing them to those fabricated from DM resin and a nanographene‐reinforced dental cast resin.^34^ Consistent with the findings of the present study, Arnold et al.^34^ found that CB dies generally exhibited superior dimensional stability and fit compared to those made from other resins. They suggested that CB resin could serve as a more reliable and potentially more environmentally sustainable alternative to the other resins tested for the AM of removable dies and corresponding partial arch dental casts.

A limitation of this study was the absence of a control group using dental stone dies, which limits direct comparison to the conventional clinical standard. This decision reflects the increasing adoption of direct digital workflows in prosthodontics, where stone dies are progressively replaced by digitally fabricated models and dies. Although commonly cited clinical thresholds for dimensional accuracy in AM casts provide a practical reference,^10^, ^13^ these thresholds are not directly comparable to stone die performance. Inclusion of a stone die control group in future studies would offer valuable clinical context and facilitate translation for clinicians and dental technicians transitioning from analog to digital workflows. The chemical composition and formulation of the CB and SB resins were not completely disclosed by the manufacturer, which restricted a comprehensive understanding of the material's properties and their influence on dimensional and positional stability. All tested resins were AM with the same printer and standardized printing and postprinting parameters. Variations in printing technologies,^9^, ^31^ printing settings,^4^, ^32^ and storage conditions^9^ could potentially influence the outcomes tested in this study. Another limitation of this study was the geometry of the tested removable dies, as variations in taper and root shapes could influence the results.^8^, ^15^, ^28^ Additionally, the design parameters of the die and corresponding cast may also impact the fit. Another limitation was the use of a single hollow cast per resin group for evaluating positional deviations. Although this approach ensured geometric consistency and minimized variability from multiple partial arch casts, it also introduced a potential source of bias, as any cast‐specific distortion could have uniformly influenced all measurements within that group. However, assessment of the partial arch casts over a 4‐week period showed a maximum deviation of 4.4 µm (SB resin, between T1 and T3), reflecting minimal impact from printing and scanning processes. Given this high dimensional stability, the use of one cast per group was considered sufficient to avoid redundant fabrication, material waste, and labor, though future studies may benefit from using multiple casts to further validate positional outcomes. The scans of all removable dies and hollow casts were performed using an IOS, which has been employed in similar studies with comparable methodologies^4^, ^8^ and is reported to have accuracy comparable to that of extraoral scanners.^40^ The use of an IOS allowed for continuous scanning of the removable dies, minimizing potential errors associated with stitching multiple scans, which can occur when using an extraoral scanner. However, the choice of IOS could affect the results.^41^, ^42^ All measurements were conducted by an experienced prosthodontist following a consistent protocol to minimize human error. However, factors such as operator technique, digitization processes, and the software used for analysis may still contribute to variability in the results. Future research should explore the effects of different printing and storage conditions on the dimensional stability and fit of removable dies made from bio‐based AM resins. Additionally, assessing the efficiency of crown contact adjustments on the tested dies would provide more insight into their clinical relevance.

## CONCLUSIONS

Within this study's limitations, the additively manufactured removable dies made from corn‐based resin mostly exhibited the highest dimensional trueness, with the crown region of the dies showing the greatest dimensional accuracy among the tested regions. These corn‐based resin dies also mostly demonstrated superior dimensional stability compared to the other materials across the different die regions and time points. Regardless of the time point, the crown region maintained the highest stability, while the base of the root region exhibited the lowest. The dimensional stability of the tested dies remained high, with minimal dimensional changes over time. In contrast, additively manufactured removable dies made from SB showed the lowest positional trueness and stability. The positional trueness and stability of the corn‐based and dental cast resin dies, when seated in their respective casts, were high and could be considered acceptable for prosthetic applications. However, the SB dies displayed deviations that exceeded acceptable limits, as defined by previously established thresholds.
